# Cross-Disciplinary Mechanistic Insights in Molecular Medicine

**DOI:** 10.3390/cimb48010019

**Published:** 2025-12-24

**Authors:** Chan-Yen Kuo

**Affiliations:** Institute of Oral Medicine and Materials, College of Medicine, Tzu Chi University, Hualien 970, Taiwan; cykuo135@gms.tcu.edu.tw

## 1. Introduction

From 2023 to 2025, the Molecular Medicine Section of *CIMB* published a series of highly cited papers that collectively reshape current views on inflammation, metabolism, cancer, and regenerative biology. These studies reflect a shift toward mechanism-driven interpretation, emphasizing how immune imbalance, metabolic reprogramming, and tissue remodeling intersect to influence disease. This editorial places these contributions in a broader context, underscoring how they refine pathogenic concepts, uncover therapeutic opportunities, and strengthen the translation of molecular discoveries into clinical practice. Together, they highlight the Section’s continued leadership in advancing precision molecular medicine.

## 2. Overview of Inflammatory Bowel Disease and Immune-Mediated Pathogenesis

A review conducted by Padoan et al. described how inflammation, autoinflammation, and autoimmunity together shape the pathogenesis of inflammatory bowel diseases (IBDs), predominantly consisting of Crohn’s disease (CD) and ulcerative colitis (UC). Padoan et al. also analyse the multi-layered immune dysregulation involving the intestinal barrier, innate immune responses, and adaptive immunity [1]. IBD arises from the interaction between genetic susceptibility, environmental exposures (exposome), microbiome alterations, and dysregulated immune responses [2]. Impairments in the mucus barrier and related protective proteins raise gut permeability, allowing microbes to cross and trigger immune activation [3]. Innate immunity plays a central role, with neutrophils, macrophages, dendritic cells, NK cells, and innate lymphoid cells contributing to cytokine-driven inflammation [4]. Neutrophil extracellular traps (NETs) are highlighted, particularly in IBD, CD, and UC [5]. Adaptive immune responses are also involved, with Crohn’s disease exhibiting a predominantly Th1/Th17-driven profile, while CD is more commonly associated with Th2-skewed immunologic activity [6]. A critical imbalance between inflammatory Th17 cells and regulatory T cells (Tregs) perpetuates disease activity [7]. Padoan et al. also emphasize autoinflammation, focusing on NOD-like receptors (NLRs) and inflammasomes (NLRP1, NLRP3, NLRC4) [1]. CD is frequently associated with loss-of-function mutations in NOD2 and impaired inflammasome activity, whereas UC more often shows gain-of-function mutations and heightened inflammasome activation [8]. In summary, the accumulating data point to autophagy regulation as a promising direction for advancing novel UC treatments [9]. On the other hand, Padoan et al. also discuss the autoimmune aspects of the disease, noting the presence of antibodies such as anti-neutrophil cytoplasm antibodies (ANCA) and anti-Saccharomyces cerevisiae antibodies (ASCA) and extraintestinal manifestations involving the joints, skin, liver, and eyes. They further highlight biomarkers, including fecal calprotectin, lactoferrin, C-reactive protein (CRP), eritrocyte sedimentation rate (ESR), and relevant autoantibodies that support diagnosis and ongoing disease assessment [10,11]. Finally, they discuss the developments in proteomics, metabolomics, and artificial intelligence that offer promising opportunities to improve diagnostic accuracy, patient classification, and treatment design [1]. This review not only compiles existing evidence but also urges a shift from conventional views toward more mechanism-based and individualized therapeutic approaches.

## 3. Hormonal Regulation of Insulin Sensitivity Across the Lifespan

Insulin resistance (IR) underpins a wide spectrum of metabolic and systemic diseases, but its manifestation and clinical impact vary distinctly between men and women [12]. Emerging evidence shows that sex-related factors such as hormones, fat distribution, genetics, and lifestyle substantially influence susceptibility to insulin resistance and its associated health effects [13]. Women generally show higher insulin sensitivity than men during their reproductive years, largely due to estrogen, which helps maintain more favorable metabolic activity across adipose tissue, the liver, pancreas, muscle, and vascular endothelium [14]. This benefit wanes after menopause, as falling estrogen promotes visceral fat gain, disrupts glucose balance, and increases risks of metabolic syndrome, type 2 diabetes, and fatty liver disease [15]. Conversely, in men, higher baseline visceral adiposity, androgen effects, and distinct metabolic profiles contribute to earlier onset and higher cardiometabolic risk [16]. Ciarambino et al. note that insulin resistance is implicated in a wide spectrum of conditions, including metabolic, cardiovascular, neurologic, and reproductive disorders, and that sex-specific factors influence these vulnerabilities. They also note that lifestyle patterns such as physical activity, diet, alcohol intake, and smoking differ between men and women, further shaping overall risk [17]. Recognizing these differences is essential for developing more precise, individualized strategies for prevention and treatment.

## 4. Challenges of CAR T-Cell Therapy in Solid Tumors and NSCLC

Chimeric antigen receptor (CAR) T-cell therapy, while transformative in hematologic malignancies, has yet to achieve comparable success in solid tumors such as non-small cell lung cancer (NSCLC) [18]. Ma et al. note that NSCLC presents several biological and microenvironmental barriers, including heterogeneous antigen expression, poor T-cell infiltration, and pronounced local immunosuppression that together limit current therapeutic efficacy [19]. Nevertheless, the ongoing development of next-generation CAR architectures and the evaluation of surface targets such as epidermal growth factor receptor (EGFR), mucin 1 (MUC1), and prostate stem cell antigen (PSCA) illustrate a steadily expanding therapeutic landscape [19,20]. Emerging antigens, including EphA2, tissue factor, and PTK7, further broaden the horizon for future NSCLC-directed CAR T-cell designs [21,22]. A notable strength of this article is its emphasis on advances in imaging technology [19]. Radionuclide imaging and reporter gene systems offer valuable real-time information on CAR T-cell location, persistence, and functional activity in vivo [23,24]. Such information is indispensable for improving dosing schemes, treatment scheduling, and combination regimens [25]. As these imaging platforms continue to evolve, they are likely to become integral to the optimization of CAR T-cell therapies for solid tumors [26].

## 5. Integrating Molecular and Clinical Perspectives in MASLD

The review by Vesković et al. offers an insightful and timely reassessment of the intricate relationship between hepatic insulin resistance and metabolic-dysfunction-associated steatotic liver disease (MASLD) [27], a perspective increasingly recognized as essential in the context of rising global metabolic disease [28]. Vesković et al. highlight that the liver actively contributes to systemic metabolic disruption, shifting traditional views of disease progression. Their analysis highlights the interplay between ER stress, UPR activation, and hepatokine signaling in perpetuating metabolic imbalance [29]. This view points to liver stress responses and circadian misalignment as underrecognized therapeutic targets. By integrating evidence across levels, the review underscores personalized cardiovascular evaluation and mutation-guided care to enhance long-term outcomes in MPNs.

## 6. Advances in Natural Polymer-Based Scaffolds for Bone Tissue Engineering

The comprehensive review by Saurav et al. offers an insightful synthesis of how natural polymers ranging from chitosan and cellulose to albumin and silk fibroin are being transformed into nano-structured scaffolds to advance bone tissue engineering [30]. Their emphasis on hierarchical organization and biomimetic design is particularly noteworthy, as these features are well documented to enhance osteoconductivity, mechanical integration, and translational applicability of scaffolds [31]. Importantly, advanced 3D printing adds further benefit by allowing precise control of pore design, gradients, and multi-material structures that better mimic natural bone architecture [32]. Additive manufacturing enables patient-specific scaffold designs with consistent micro- to nanoscale features, enhancing the translational potential of natural polymer scaffolds and supporting their broader use in regenerative medicine [33].

## 7. Dual Roles of NGF in Inflammation and Tumor Progression

Nerve growth factor (NGF), known historically for its neurobiological relevance, is gaining recognition as a pivotal factor that bridges immune-driven inflammation and malignant transformation [34]. Ferraguti et al. highlight the dual roles of NGF, showing that it can support tumor growth, angiogenesis, and immune escape, yet in other contexts induce apoptosis and help limit inflammation [35]. This apparent paradox underscores a core concept in cancer biology: molecular signals act through dynamic interactions within the tumor microenvironment, jointly shaping how the disease evolves [36]. A major insight is NGF’s regulatory influence in the tumor microenvironment, where it affects macrophage function, cytokine signaling, epithelial–mesenchymal transition, and the maintenance of stem-like cancer cells. [37,38]. These linked pathways help explain NGF’s tumor-promoting roles and highlight its potential as a therapeutic target, particularly as precision medicine increasingly focuses on pathway-directed interventions.

## 8. UV-Induced Genomic Imprinting and Early Events in Skin Carcinogenesis

The review by Al-Sadek and Yusuf provides an incisive synthesis of how ultraviolet (UV) radiation imprints the genome long before cutaneous malignancies emerge [39]. As noted in classic photobiology research, UV exposure is a mutational force woven into the skin’s biology [40], a concept reinforced by the authors’ detailed discussion of cyclobutene pyrimidine dimers (CPDs), 6-4 photoproducts (6-4PPs), and the increasingly recognized dark CPDs [41]. Their integration of pigment biology echoes earlier findings demonstrating that pheomelanin-associated ROS amplify melanoma susceptibility [42]. A key point is the move toward active photoprotection, as photolyase-based topicals and *Polypodium leucotomos* extracts aim to boost cellular DNA repair instead of relying solely on passive UV blocking [43]. The review also points to advances in AI-driven diagnostic tools, noting that machine-learning methods increasingly outperform conventional visual evaluation in early skin cancer detection, consistent with findings from other studies [44,45]. Overall, the article emphasizes that progress in skin cancer research depends on integrating mechanistic insights with technological advances, a partnership already reshaping prevention, diagnosis, and therapy.

## 9. Mitochondrial Dysfunction as a Central Driver of Brain Aging and Neurodegeneration

Mitochondrial biology has re-emerged as a unifying framework for understanding brain aging and neurodegeneration, and the review by Bartman et al. provides a timely synthesis of this rapidly evolving field [46]. The review connects mitochondrial defects such as diminished oxidative phosphorylation (OXPHOS) activity, mtDNA buildup, and impaired mitophagy to wider impacts, including cognitive decline, persistent inflammation, and heightened neuronal susceptibility. Their analysis underscores that mitochondria serve as both energy hubs and immune modulators, as mtDNA, ROS, cardiolipin, and other mitochondrial damage-associated molecular patterns (DAMPs) can activate inflammatory pathways including NLRP3 and cyclic GMP-AMP synthase (cGAS)-stimulator of interferon genes (STING) [47,48,49]. This perspective reinforces a broader concept: neurodegeneration arises not from a single insult but from converging failures in metabolism, quality control, and inflammatory regulation [50]. Equally noteworthy is the translational perspective, as strategies like caloric restriction, exercise, mitochondrial-directed antioxidants, and mitophagy-boosting agents continue to demonstrate potential in preclinical studies [51]. As precision therapeutics advance, strategies aimed at restoring mitochondrial resilience may ultimately reshape how we prevent and treat age-related brain disorders [52].

## 10. Molecular Drivers Shaping the Pathogenesis and Progression of PDAC

The review by Stefanoudakis et al. offers a concise and authoritative overview of the molecular architecture underlying pancreatic ductal adenocarcinoma (PDAC), emphasizing the pivotal contributions of TP53, CDKN2A, SMAD4, and KRAS to disease progression and therapeutic resistance [53]. The authors show that TP53-mediated microenvironmental changes and SMAD4 loss within altered TGF-β signaling promote tumor invasion and metastasis, aligning with prior genomic and functional evidence [54]. Their discussion on CDKN2A inactivation and KRAS-addicted metabolic reprogramming further underscores why PDAC remains one of the most lethal malignancies [55]. As precision oncology evolves, integrating these molecular signatures into early detection, risk stratification, and targeted therapeutic development becomes increasingly essential. This review not only consolidates current evidence but also defines promising avenues for translational research aimed at improving clinical outcomes in PDAC.

## 11. Genetic and Inflammatory Drivers of Thrombotic Risk in MPNs

The narrative review by Todor et al. provides an insightful synthesis of how Philadelphia-negative myeloproliferative neoplasms (MPNs), including polycythemia vera, essential thrombocythemia, and primary myelofibrosis, predispose patients to substantial cardiovascular morbidity [56]. The authors outline how mutations like JAK2 V617F, MPL, and CALR heighten thrombotic risk by driving chronic inflammation, endothelial activation, and abnormal leukocyte–platelet interactions [57]. Particularly compelling is the discussion of cytokine dysregulation, where elevated IL-1β, IL-6, and TNF-α reinforce clonal expansion while accelerating atherosclerosis and vascular stiffness [58]. They further point to newer mechanisms, including NET formation, RAS imbalance, and microRNA alterations that may contribute to the heightened cardiovascular risk seen in MPNs. By integrating evidence across molecular, cellular, and clinical levels, the review emphasizes the importance of personalized cardiovascular evaluation and mutation-guided management to enhance long-term patient outcomes [43].

## Data Availability

Not applicable.
